# Complete asymmetric polarization conversion at zero-eigenvalue exceptional points of non-Hermitian metasurfaces

**DOI:** 10.1515/nanoph-2024-0391

**Published:** 2024-10-31

**Authors:** Donghak Oh, Soojeong Baek, Sangha Lee, Kyungmin Lee, Jagang Park, Zhaowei Liu, Teun-Teun Kim, Bumki Min

**Affiliations:** Department of Physics, Korea Advanced Institute of Science and Technology (KAIST), Daejeon 305-751, Republic of Korea; Department of Electrical and Computer Engineering *,* University of California, San Diego, 9500 Gilman Drive, La Jolla, CA 92093, USA; Department of Mechanical Engineering, Korea Advanced Institute of Science and Technology (KAIST), Daejeon 305-751, Republic of Korea; Department of Electrical Engineering and Computer Sciences, University of California, Berkeley, CA, USA; Department of Physics, 35029 University of Ulsan, Ulsan 44610, Republic of Korea

**Keywords:** non-Hermitian metasurfaces, exceptional points, polarization conversion, gain-incorporated metasurface, circular dichroism

## Abstract

Non-Hermitian systems can be tuned to exhibit exceptional points, where both eigenvalues and eigenstates coalesce concurrently. The inherent adaptability of photonic non-Hermitian systems in configuring gain and loss has allowed us to observe a plethora of counterintuitive phenomena, largely as a consequence of the eigenspace reduction at these exceptional points. In this work, we propose a non-Hermitian metasurface that, through the incorporation of gain, enables complete asymmetric polarization conversion at an exceptional point with a zero eigenvalue. Specifically, we provide numerical evidence for this concept by designing a non-Hermitian metasurface that facilitates polarization conversion from right to left circular polarization, while preventing conversion in the reverse direction and co-polarized transmission. Furthermore, our investigation reveals that this specific form of complete asymmetric polarization conversion results in maximum circular dichroism in transmission, thereby eliminating the need for external chirality or three-dimensional helical structures. This non-Hermitian technique offers an intriguing approach to designing polarization-sensitive optical devices and systems, further expanding their functionalities and capabilities.

## 1 Introduction

The chiro-optical effects are characterized by distinct optical responses that depend on the handedness of the incident circularly polarized light and the chirality of the medium through which it passes [1], [2], [3], [4]. These effects originate from the unique coupling of electric and magnetic fields in naturally occurring or artificially engineered chiral materials. This coupling results in distinct optical behaviors for right- and left-circularly polarized light (RCP and LCP), effectively lifting the degeneracy typically seen in non-chiral environments. Given the relatively weak chiro-optical effects in natural materials, extensive research has focused on the development of three-dimensional (3D) artificial helical structures. These structures are designed to enhance the mutual coupling between electric and magnetic fields, aiming to enhance the chiro-optical responses [5], [6], [7], [8], [9]. Simultaneously, planar chiral structures, which feature broken in-plane mirror symmetry, have been employed to achieve strong chiro-optical responses [10], [11], [12], [13]. Owing to their planar configuration, these structures are more easily fabricated using conventional lithographic techniques, making them particularly advantageous for various applications. A notable application of planar chiral structures is asymmetric polarization conversion (APC), where one circular polarization is efficiently converted to its opposite, while the conversion in the opposite direction is significantly less effective [14], [15], [16].

Recent advancements in non-Hermitian physics have significantly enhanced our understanding of polarization manipulation through artificial structures. These developments have expanded the possibilities for their design by leveraging insights into the interactions within open environments [17], [18], [19], [20], [21], [22]. It is now well-recognized that non-Hermitian photonic systems can be tailored to feature exceptional points (EPs), specifically those where polarization eigenstates coalesce. This coalescence at the EPs reduces the dimensionality of the polarization eigenspace, leading to the emergence of unique wave phenomena that are dependent on polarization. Over the past decade, the exploration of non-Hermitian polarization degeneracy has spanned various photonic systems, including photonic crystal slabs [23], [24], [25], [26], planar microcavities [27], [28], and metasurfaces [17], [18], [19], [20], [22], [29], [30]. Among these, non-Hermitian metasurfaces are particularly notable for their study of novel polarization effects, due to the versatility of their materials and meta-atom designs. Our recent work has demonstrated active polarization control using a THz non-Hermitian metasurface, which consists of an array of coupled split-ring resonators with an integrated gated graphene micro-ribbon [31]. By adjusting the frequency of the incident wave and the gate voltage applied to the graphene, we accessed the chiral EP to explore the narrowed polarization eigenspace and its impact at the output. However, for a comprehensive understanding of polarization behavior at the EP, it is crucial to examine not only the coalesced polarization eigenstate but also the eigentransmission.

In this work, we introduce a gain-incorporated non-Hermitian metasurface designed to harness both the zero eigenvalue and the coalesced circularly polarized eigenstates at a chiral EP, thereby enabling complete asymmetric polarization conversion (CAPC). Specifically, we consider a situation where near-complete polarization conversion from right to left circularly polarized states is achieved, while the conversion in the opposite direction and co-polarized transmission are simultaneously blocked at the chiral EP. Our analysis begins with the application of temporal coupled-mode theory (TCMT) to determine the conditions necessary for observing CAPC at a chiral EP with a zero eigenvalue. The viability of this approach is further confirmed through numerical simulations of a non-Hermitian metasurface, consisting of an array of coupled lossy meta-atoms arranged on a substrate with gain. We further demonstrate that CAPC results in maximal circular dichroism in transmission (CDT), revealing an intriguing aspect of the eigenvalue-dependent polarization phenomenon at chiral EPs.

## 2 Results and discussion

### 2.1 CAPC at chiral zero-eigenvalue EPs

First, we examine the Jordan form of a non-Hermitian Jones matrix **J** at the EP, which can be expressed using a coalesced polarization eigenstate $\left|\sigma \right\rangle$ and a Jordan vector $\left|\mu \right\rangle$ . These vectors satisfy the conditions $\left(J-t_{e}I\right)\left|\mu \right\rangle =\left|\sigma \right\rangle$ and $\left\langle \mu \left|\sigma \right\rangle \right.=0$ , where **I** is the 2 × 2 identity matrix [32], [33]. The Jones matrix can be represented as,

$$J=t_{e}\left|\sigma \right\rangle \left\langle \sigma \right|+t_{\sigma \mu }\left|\sigma \right\rangle \left\langle \mu \right|+t_{e}\left|\mu \right\rangle \left\langle \mu \right|,$$

where *t*_*σμ*_ and *t*_*e*_ denote the cross-polarized transmission from $\left|\mu \right\rangle$ to $\left|\sigma \right\rangle$ and the degenerate eigenvalue (or eigentransmission), respectively. The Jordan form at the EP inherently signifies APC with conditions *t*_*μσ*_ = 0, *t*_*σμ*_ ≠ 0 and equal co-polarized transmissions (*t*_*e*_ = *t*_*σσ*_ = *t*_*μμ*_). Furthermore, this Jordan form indicates that at the EPs, the output polarization can be expressed as a weighted sum of the coalesced eigenstate and the associated Jordan vector, provided the eigentransmission is non-zero. However, when the eigentransmission becomes zero, only the polarization component parallel to the coalesced eigenstate appears at the output.

**Figure 1:**
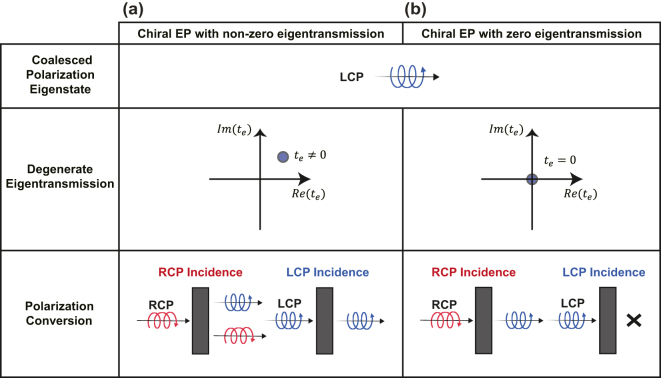
Eigentransmission-dependent polarization conversion at chiral EPs. (a) Asymmetric polarization conversion (APC) at the chiral nonzero-eigenvalue EP, (b) complete asymmetric polarization conversion (CAPC) at the chiral zero-eigenvalue EP.

Figure 1 illustrates the dependence of polarization conversion (PC) on eigentransmission at chiral EPs. When the eigentransmission at the chiral EP is non-zero (*t*_*e*_ ≠ 0, see Figure 1(a)) in the circular polarization basis ( $\left|\sigma \right\rangle =\left|L\right\rangle$ and $\left|\mu \right\rangle =\left|R\right\rangle$ ), APC is observed alongside non-zero co-polarized transmission (e.g., *t*_*RL*_ = 0, *t*_*LR*_ ≠ 0, and *t*_*RR* =_ *t*_*LL*_ ≠ 0_)_. In contrast, when the eigentransmission at the chiral EP is zero (*t*_*e*_ = 0, see Figure 1(b)), CAPC occurs without co-polarized transmission (*t*_*RL*_ = 0, *t*_*LR*_ ≠ 0, and *t*_*RR*_ = *t*_*LL*_ = 0), as suggested by the simplified Jones matrix $J=t_{LR}\left|R\right\rangle \left\langle L\right|$ . In the subsequent section, we propose a non-Hermitian metasurface situated on a gain substrate as a potential platform for realizing CAPC at the chiral zero-eigenvalue EP.

### 2.2 Conditions for achieving CAPC

**Figure 2:**
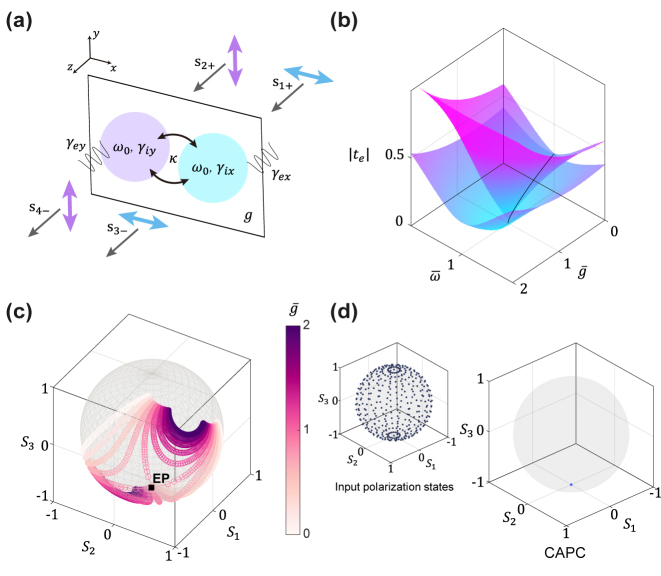
Analytic modeling of gain-incorporated non-Hermitian metasurfaces. (a) Schematic illustration of a gain-incorporated non-Hermitian metasurface consisting of two coupled resonators on a gain substrate. Two coupled resonators (drawn with cyan and magenta colors) are assumed to be connected to four ports. Two incoming wave amplitudes *s*_1+_ (*x*-polarization) and *s*_2+_ (*y*-polarization) and two outgoing wave amplitudes *s*_3−_ (*x*-polarization) and *s*_4−_ (*y*-polarization), which are relevant to the evaluation of the Jones matrix, are shown. (b) Eigentransmission amplitude plotted in a two-dimensional parameter space spanned by normalized incident frequency ( $\bar{\omega }=\omega /\omega_{EP}$ ) and normalized gain rate ( $\bar{g}=g/g_{EP}$ ), where *ω*_*EP*_ and *g*_*EP*_ represent the incident angular frequency and gain rate at the chiral EP, respectively. (c) Polarization eigenstates are plotted on a Poincaré sphere as a function of $\bar{\omega }$ and $\bar{g}$ , with color shading used to denote different gain rates. Frequency-dependent polarization eigenstates are represented by scatters of the same color. (d) Uniformly distributed input polarization states mapped on Poincaré sphere (left). All input polarization states are converted to the LCP state at the output.

To illustrate a viable approach for realizing CAPC, we examine a non-Hermitian metasurface with unit cells composed of two coupled resonators, situated on a gain substrate. The two constituent resonators are assumed to have the same resonance frequency of *ω*_0_/2*π*, with their major axes aligned along the *x*- and *y*-directions, respectively (see Figure 2(a)). Using temporal coupled-mode theory (TCMT) [22], [34], the non-Hermitian Jones matrix can be expressed in the linear polarization basis as follows:

$$T_{l}=\xi I+\eta \left[\begin{array}{cc} \Omega_{y}+j\Gamma & K\\ K & \Omega_{x}-j\Gamma \end{array}\right].$$

To simplify the expression, we introduce the following dimensionless parameters: $\Omega_{x}=\left(\omega_{x}-\omega \right)/\gamma_{ex}$ , $\Omega_{y}=\left(\omega_{y}-\omega \right)/\gamma_{ey}$ , $\Gamma =\left(\gamma_{iy}-g\right)/2\gamma_{ey}-\left(\gamma_{ix}-g\right)/2\gamma_{ex}$ , and $K=-\kappa /\sqrt{\gamma_{ex}\gamma_{ey}}$ . Here, *ω* represents the angular frequency of the incident light, while *κ*, *g*, *γ*_*eμ*_ and *γ*_*iμ*_ denote the coupling, gain, radiative loss, and intrinsic loss rates, respectively $\left(\mu =x,y\right)$ . In the analysis, two coupled resonators are connected to four ports: two ports describe *x*- and *y*-polarized waves on the input side of the metasurface, while the remaining two ports describe the same waves on the output side (Figure 2(a)). The incoming and outgoing waves are represented by $\left|s_{+}\right\rangle ={\left(s_{1+},s_{2+},s_{3+},s_{4+}\right)}^{T}$ and $\left|s_{-}\right\rangle ={\left(s_{1-},s_{2-},s_{3-},s_{4-}\right)}^{T}$ , respectively. To derive the Jones matrix, we analytically establish the relationship between the incoming wave amplitudes *s*_1+_ (*x*-polarization) and *s*_2+_ (*y*-polarization), and the outgoing wave amplitudes *s*_3−_ (*x*-polarization) and *s*_4−_ (*y*-polarization) (see Supplementary Information for details). The two constituent resonators are assumed to have different radiative loss rates (*γ*_*ex*_ ≠ *γ*_*ey*_), while experiencing equal influence from the gain substrate. Accounting for the substrate gain, the net intrinsic loss rates of the two resonators are modified to *γ*_*ix*_ − *g* and *γ*_*iy*_ − *g*, respectively.

By solving the matrix eigenvalue equation, $T_{l}\left|\psi_{e}\right\rangle =t_{e}\left|\psi_{e}\right\rangle$ , we can obtain the eigentransmissions and the corresponding polarization eigenstates. Examining the eigentransmissions reveals a pair of chiral EPs. One EP has a coalesced LCP eigenstate, characterized by the condition Ω_*x*_ = Ω_*y*_ = Ω and Γ = −*K*. The other EP has a coalesced RCP eigenstate, characterized by Ω_*x*_ = Ω_*y*_ = Ω and Γ = *K*. Without loss of generality, in this work, we primarily focus on the chiral EP characterized by a coalesced LCP eigenstate. At this specific chiral EP, the eigentransmission is given by *t*_*e*_ = *ξ* + *η*Ω, and the coalesced eigenstate is represented by $\left|\psi_{e}\right\rangle =\frac{1}{\sqrt{2}}{\left[\begin{array}{cc} 1 & j \end{array}\right]}^{T}$ (see Supplementary Information for details).

From the eigentransmission expression, it can be shown that the following relationships between a set of parameters are sufficient to ensure zero eigentransmission at the chiral EP (see Supplementary Information):*ω* = *ω*_0_ = *ω*_*EP*_ and $\gamma_{ex}/\gamma_{ey}=-\left(\gamma_{ix}-g\right)/\left(\gamma_{iy}-g\right)=\delta >0$ . The latter requirement indicates that the gain rate should be adjusted such that the net intrinsic loss rates, *γ*_*ix*_ − *g* and *γ*_*iy*_ − *g*, have opposite signs. This can be achieved if the two resonators are composed of materials with different intrinsic loss rates (*γ*_*ix*_ ≠ *γ*_*iy*_), and the gain rate is set to a value between *γ*_*ix*_ and *γ*_*iy*_. To illustrate this, we plot the eigentransmission amplitude in a two-dimensional parameter space (Figure 2(b)), defined by the normalized incident frequency ( $\bar{\omega }=\omega /\omega_{EP}$ ) and the normalized gain rate ( $\bar{g}=g/g_{EP}$ ). Here, *ω*_*EP*_ and *g*_*EP*_ represent the incident angular frequency and gain rate at the chiral EP, respectively. In this example, the following parameters are used to model the non-Hermitian metasurface: *ω*_*EP*_/2*π* = 193 THz, *γ*_*ex*_/*ω*_*EP*_ = 0.06, *γ*_*ey*_/*ω*_*EP*_ = 0.1, *γ*_*ix*_/*ω*_*EP*_ = 0.02, *γ*_*iy*_/*ω*_*EP*_ = 0.04, *κ*/*ω*_*EP*_ = 0.00968, and *g*_*EP*_/*ω*_*EP*_ = 0.0275. As shown in Figure 2(b), the eigentransmission approaches zero at the chiral EP, specifically at $\left(\begin{array}{cc} \bar{\omega }, & \bar{g} \end{array}\right)=\left(1,1\right)$ . The polarization eigenstates are plotted on a Poincaré sphere as a function of $\bar{\omega }$ and $\bar{g}$ , with color shading indicating different gain rates to enhance visualization (Figure 2(c)). As the parameters approach the chiral EP, the paired polarization eigenstates progressively coalesce into the LCP state located at the south pole of the Poincaré sphere [31]. The CAPC at the chiral zero-eigenvalue EP is further verified by observing that all output polarization states converge at the south pole of the Poincaré sphere for uniformly distributed input polarization states (Figure 2(d)).

### 2.3 Numerical verification of CAPC

**Figure 3:**
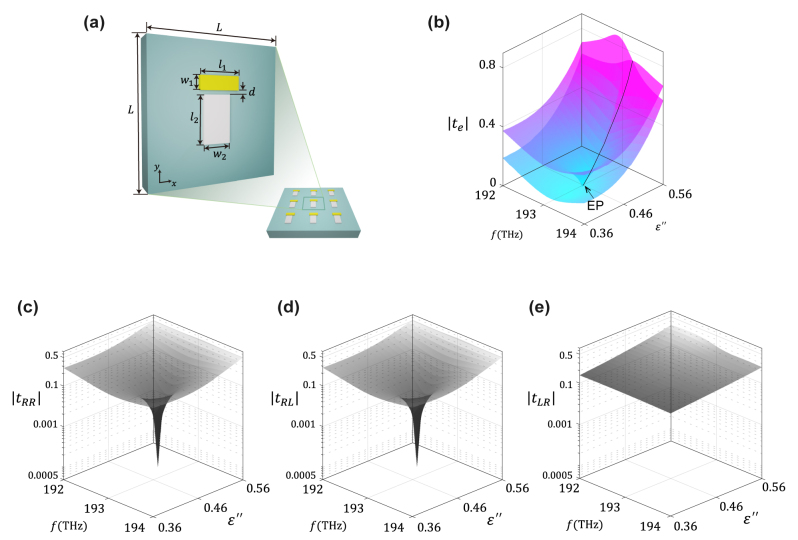
Design of the gain-incorporated non-Hermitian metasurface for CAPC. (a) Schematic illustration of the gain-incorporated non-Hermitian metasurface, with the geometric dimensions of the unit cell determined to be ***L*** = 600 nm, ***l***_1_ = 193 nm, ***l***_2_ = 197 nm, ***w***_1_ = 82 nm, ***w***_2_ = 121 nm, and ***d*** = 60 nm. (b) Numerically extracted eigentransmission magnitude plotted in the parameter space spanned by the input frequency (***f***) and the imaginary part of substrate permittivity (***ɛ*″**). (c–e) Numerically extracted transmission amplitudes $\left|t_{LR}\right|$ , $\left|t_{RL}\right|$ , and $\left|t_{RR}\right|=\left|t_{LL}\right|$ in a two-parameter space spanned by ***f*** and ***ɛ*″**.

To demonstrate the feasibility of gain-assisted ACPC at the chiral zero-eigenvalue EP, we designed a non-Hermitian metasurface. This metasurface consists of two coupled plasmonic nano-rod resonators placed on a gain substrate (Figure 3(a)). Each of the two plasmonic nano-rod resonators, oriented with their major axes perpendicular to each other, is designed to resonate at the same angular frequency, *ω*_0_, when exposed to incident light polarized along their respective major axes. By varying the geometrical parameters of the resonators and the lattice constant, we can adjust the ratio, *δ*, between the radiative loss rates. To achieve distinct intrinsic loss rates, two types of plasmonic nano-rods can be utilized: one made of silver and the other made of gold. Specifically, the Drude model, which describes the relative permittivity of metals, was employed in our simulations with the following parameters: a plasma frequency *ω*_*p*_ = 1.2052 × 10^16^ rad/s and a collision frequency *γ* = 1.3697 × 10^14^ rad/s for gold, and *ω*_*p*_ = 1.1454 × 10^16^ rad/s and *γ* = 1.1938 × 10^14^ rad/s for silver [35]. In the simulations conducted using a finite element method solver, the following geometrical parameters were employed (see Figure 3(a)): *L* = 600 nm, *l*_1_ = 193 nm, *l*_2_ = 197 nm, *w*_1_ = 82 nm, *w*_2_ = 121 nm, and *d* = 60 nm. The coupling between the resonators is tuned by adjusting their relative positions within the unit cell. As discussed in the previous section, by fine-tuning the gain rate and the frequency of the incident wave, we can approach the chiral zero-eigenvalue EP and thereby observe CAPC. In numerical simulations, the change in gain rate is modeled by varying the imaginary part of the substrate permittivity. The complex-valued permittivity, *ɛ* = *ɛ*′ − *jɛ*″, is specifically modeled after an InGaAsN/InP quantum well [36], [37], with the real part of the permittivity (*ɛ*′ = 9.5), held approximately constant, while the imaginary part (*ɛ*″) is varied [38], [39].

To confirm the presence of a chiral zero-eigenvalue exceptional point (EP), we performed numerical calculations of the eigentransmission for the designed non-Hermitian metasurface. These calculations were conducted in a two-parameter space defined by the input frequency and the imaginary part of the permittivity (Figure 3(b)). The eigentransmission reveals the topology of a self-intersecting Riemann surface, identifying the chiral EP of a nearly zero eigenvalue at approximately $\left(f_{EP},\varepsilon_{EP}^{\prime \prime \prime }\right)$ = (193.08 THz, 0.4685). Additionally, the Jones matrix elements are extracted and plotted in the two-parameter space to substantiate the fulfillment of the CAPC condition. In Figure 3(c–e), it is evident that the cross-polarized transmission *t*_*RL*_ and the co-polarized transmissions *t*_*RR*_ and *t*_*LL*_ sharply approach zero at the chiral zero-eigenvalue EP, whereas the other cross-polarized transmission *t*_*LR*_ remains nearly constant. It is worth noting that at the chiral zero-eigenvalue EP, the intensity conversion efficiency of the proposed metasurface, initially estimated to be approximately ${\left|t_{LR}\right|}^{2}\approx 4\;\%$ , can be significantly enhanced to up to 25 % by optimizing the geometrical parameters of the unit cell. In addition to examining the transmission amplitude, understanding the system behavior near the chiral EP requires a thorough analysis of the transmission phase spectra. The phase spectra offer crucial insights into the underlying dynamics, particularly in the vicinity of EPs, where rapid phase variations occur. These variations reflect the topological characteristics of the EP, manifesting as pronounced phase changes in its proximity. To further illustrate this phenomenon, the transmission phase spectra and their winding behavior across the two-parameter space are presented in Figure S2 of the Supplementary Information.

### 2.4 Maximal CDT at chiral zero-eigenvalue EPs

**Figure 4:**
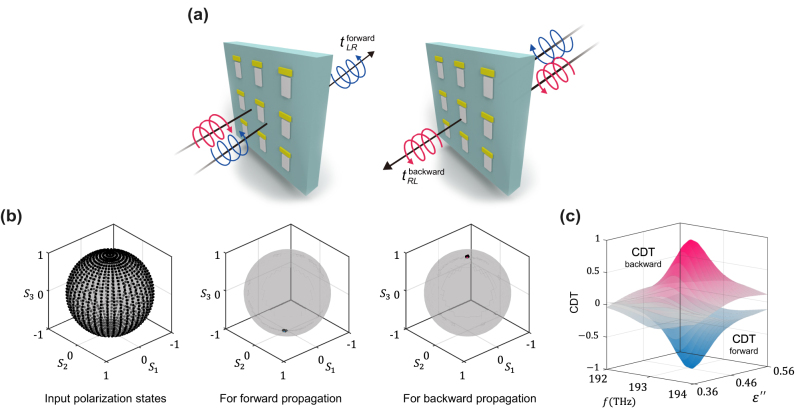
Direction-dependent CAPC and maximal CDT at the chiral zero-eigenvalue EP. (a) Schematic illustration of direction-dependent CAPC in gain-incorporated non-Hermitian metasurfaces. (b) For incidence of the homogenous input state mapped on Poincaré sphere, the LCP and RCP components are mostly transmitted for forward and backward propagation, respectively. Uniformly distributed input polarization states mapped on Poincaré sphere (left). All input polarization states are converted to the LCP state at the output. (c) Numerically-extracted CDT in a two-parameter space spanned by ***f*** and ***ɛ*″**. **CDT** forward (blue surface) and **CDT** backward (red surface) for forward and backward propagation, respectively.

The proposed metasurface interacts with waves in a direction-dependent manner, effectively altering the polarization of light differently based on whether it is incident from the front or the back. This phenomenon is attributed to the inherent planar chirality, arising from the broken in-plane mirror symmetry of the unit cell. In particular, CAPC occurs when an LCP wave is incident on the front surface of the metasurface. Conversely, CAPC is observed with an RCP wave when it is incident from the rear. This indicates that direction-dependent CAPC can be achieved at the chiral zero-eigenvalue EP (Figure 4(a)). To demonstrate how the metasurface converts circularly polarized waves based on the incident direction, we performed simulations exposing the metasurface to all possible states of polarized waves at the chiral zero-eigenvalue EP (Figure 4(b)). In the case of forward incidence, the output polarization states converge at the south pole (LCP), whereas for backward incidence, they converge at the north pole (RCP). The output polarization states are tightly clustered around the poles, revealing an extreme case of non-unitary polarization transformation at the chiral zero-eigenvalue EP.

Notably, CAPC maximizes circular dichroism in transmission (CDT), defined as the normalized difference in transmission between left-handed and right-handed circularly polarized light [40], [41],

$$\text{CDT}=\frac{\left({\left|t_{RL}\right|}^{2}+{\left|t_{LL}\right|}^{2}\right)-\left({\left|t_{LR}\right|}^{2}+{\left|t_{RR}\right|}^{2}\right)}{\left({\left|t_{RL}\right|}^{2}+{\left|t_{LL}\right|}^{2}\right)+\left({\left|t_{LR}\right|}^{2}+{\left|t_{RR}\right|}^{2}\right)}$$

For the simulated case, all transmission amplitudes except for *t*_*LR*_ approach nearly zero at the chiral zero-eigenvalue EP for forward incidence. Consequently, CDT approaches a value of −1, indicating its maximum magnitude (Figure 4(c)). For backward incidence, the value of CDT is inverted, approaching +1 at the chiral zero-eigenvalue EP. Through numerical simulations, we achieve blocking of RCP at approximately 50 dB while maximizing the CDT by simultaneously suppressing co-polarized transmission. This enhanced level of polarization control is not simply a result of parameter optimization; it arises from mathematically rigorous conditions.

## 3 Conclusions

In this work, we propose a viable solution for achieving CAPC by utilizing a chiral EP with zero eigentransmission in a gain-incorporated non-Hermitian metasurface. Our findings demonstrate that such a non-Hermitian metasurface can facilitate the conversion from right to left circular polarization (and vice versa), while simultaneously blocking both the reverse conversion and co-polarized transmission. It is noteworthy that CAPC in the circular polarization basis was first discovered using multilayer polarizers [42]. In contrast, our method distinguishes itself by achieving CAPC with a single-layer structure, thereby eliminating the need for multiple optical components. This unique form of polarization manipulation also results in maximal CDT, highlighting the potential of non-Hermitian systems in enhancing the functionality of optical devices. Notably, this method circumvents the need for external chirality or complex three-dimensional helical (or multilayered) structures traditionally required to enhance CDT. Additionally, this work sheds light on the eigenvalue-dependent phenomena at the EP, drawing a mathematical parallel to the coherent perfect absorption observed at the zero-eigenvalue EP in two-coupled microcavities [43]. The implications of our non-Hermitian metasurface offer new avenues for designing polarization-sensitive devices and systems with expanded capabilities.

## Supplementary Material

Supplementary Material Details
