# Supplementary material for: Complete asymmetric polarization conversion at zero-eigenvalue exceptional points of non-Hermitian metasurfaces
Source: Nanophotonics. 2024 Oct 31;13(24):4409–16. doi: 10.1515/nanoph-2024-0391 (PMC11636506; doi:10.1515/nanoph-2024-0391)
Supplement: Supplementary file 1 — Supplementary Material Details [file j_nanoph-2024-0391_suppl_001.docx]

Supplementary Information for

**Complete asymmetric polarization conversion at zero-eigenvalue exceptional points** **of non-Hermitian metasurfaces**

## Donghak Oh^1,†^, Soojeong Baek^2,†^, Sangha Lee^3,†^, Kyungmin Lee^1^, Jagang Park^4^, Zhaowei Liu^2^, Teun-Teun Kim^5^, and Bumki Min^1,3*^

*^1^Department of Physics, Korea Advanced Institute of Science and Technology (KAIST), Daejeon 305-751, Republic of Korea*

*^2^Department of Electrical and Computer Engineering, University of California, San Diego, 9500 Gilman Drive, La Jolla, CA, 92093 USA ^3^Department of Mechanical Engineering, Korea Advanced Institute of Science and Technology (KAIST),* Daejeon 305-751, Republic of Korea *^4^Department of Electrical Engineering and Computer Sciences, University of California, Berkeley, CA, USA*

*^5^Department of Physics, University of Ulsan, Ulsan 44610, Republic of Korea*

^†^ *Donghak Oh, Soojeong Baek and Sangha Lee contributed equally to this work.*

* *Corresponding author: bmin@kaist.ac.kr*

**
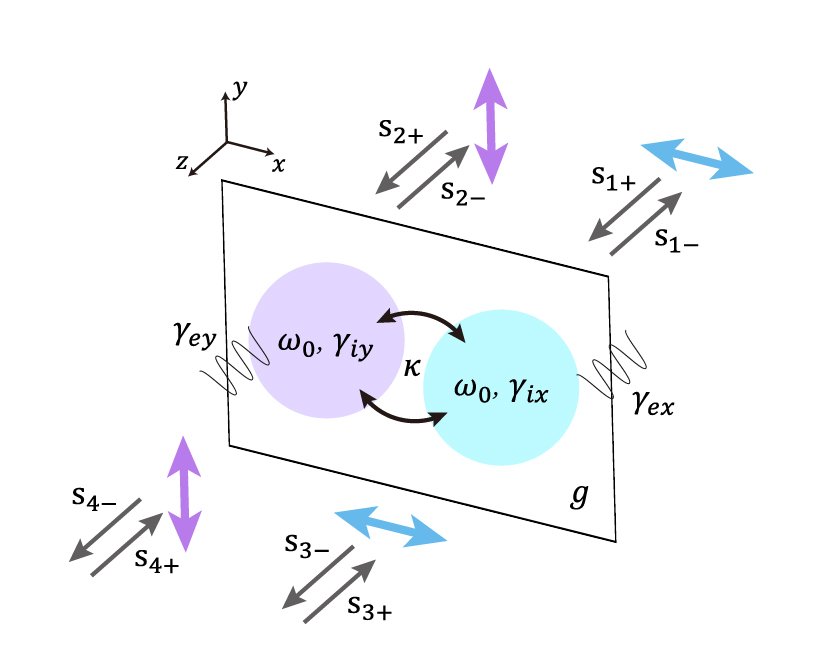
Derivation of the jones matrix for gain-incorporated non-Hermitian metasurfaces**

**Figure S1.** Schematic illustration of a system comprising coupled resonators on a gain substrate.

We derived the Jones matrix for the gain-incorporated non-Hermitian metasurface using temporal coupled-mode theory (TCMT)^1–5^. For this purpose, we considered an anisotropic metasurface situated in the *x*-*y* plane, with waves propagating along the positive and negative *z*-axis^6^ (Fig. S1). The *x*- and *y*-directional electric dipole resonances are described by the modal amplitude vector $\left. \left| a \right. \right\rangle={(a_{x},a_{y})}^{T}$, assuming harmonic time dependence of $e^{j\omega t}$. The two-mode resonator system is coupled to the four ports associated with the incoming and outgoing waves, denoted as $\left. \left| s_{+} \right. \right\rangle={(s_{1+},s_{2+}, s_{3+},s_{4+})}^{T}$ and $\left. \left| s_{-} \right. \right\rangle={(s_{1-},s_{2-}, s_{3-},s_{4-})}^{T}$, respectively. The temporal coupled-mode equations can then be formulated as follows:

$$\frac{d}{dt}\left. \left| a \right. \right\rangle=\left( j\boldsymbol{\Omega}-\boldsymbol{\Gamma}_{\boldsymbol{e}}-\boldsymbol{\Gamma}_{\boldsymbol{i}} \right)\left. \left| a \right. \right\rangle+\mathbf{K}^{\mathbf{T}}\left. \left| s_{+} \right. \right\rangle,$$

$$\left. \left| s_{-} \right. \right\rangle=\mathbf{C}\left. \left| s_{+} \right. \right\rangle+\mathbf{D}\left. \left| a \right. \right\rangle,$$

where the matrices are:

$\boldsymbol{\Omega}=\left[ \begin{matrix} \omega_{x} & \kappa\\ \kappa& \omega_{y} \end{matrix} \right], \boldsymbol{\Gamma}_{\boldsymbol{e}}\boldsymbol{=}\left[ \begin{matrix} \gamma_{ex} & 0 \\ 0 & \gamma_{ey} \end{matrix} \right]\boldsymbol{,}$ $\boldsymbol{\Gamma}_{\boldsymbol{i}}\boldsymbol{=}\left[ \begin{matrix} \gamma_{ix}-g & 0 \\ 0 & \gamma_{iy}-g \end{matrix} \right]$**.**

Here, $\omega_{\mu}$, $\kappa$, $\gamma_{e\mu}$, $\gamma_{i\mu}$, and $g$ are the (angular) resonance frequency, coupling, radiative and intrinsic loss and gain rates, respectively ($\mu=x, y$). The matrix **C** represents the direct scattering of the incoming waves to the outgoing waves without interacting with the resonator modes. The matrices $\mathbf{K}^{\mathbf{T}}$ **and** $\mathbf{D}$ represent the coupling between the resonator modes and the input ports, and the resonator modes and the output ports, respectively^1^. Considering energy conservation and time-reversal symmetry, we can derive the matrices $\mathbf{C}$, $\mathbf{K}$ and $\mathbf{D}$ as follows:

$$\mathbf{C}=\left[ \begin{matrix} \begin{matrix} 0 & 0 \\ 0 & 0 \end{matrix} & \begin{matrix} 1 & 0 \\ 0 & 1 \end{matrix} \\ \begin{matrix} 1 & 0 \\ 0 & 1 \end{matrix} & \begin{matrix} 0 & 0 \\ 0 & 0 \end{matrix} \end{matrix} \right], \mathbf{D=}\mathbf{K}=\left[ \begin{matrix} \begin{matrix} j\sqrt{\gamma_{ex}} & 0 \\ 0 & j\sqrt{\gamma_{ey}} \end{matrix} \\ \begin{matrix} j\sqrt{\gamma_{ex}} & 0 \\ 0 & j\sqrt{\gamma_{ey}} \end{matrix} \end{matrix} \right].$$

It follows that the scattering matrix $\mathbf{S}$ of the system, which describes the relationship between the amplitudes of the incoming and outgoing waves, is given by:

$$\mathbf{S}=\mathbf{C}-\mathbf{D}\mathbf{H}^{\boldsymbol{-1}}\mathbf{D}^{\mathbf{T}}\boldsymbol{=}\left[ \begin{matrix} \mathbf{R}_{lf} & \mathbf{T}_{lb} \\ \mathbf{T}_{lf} & \mathbf{R}_{lb} \end{matrix} \right]\boldsymbol{=}\left[ \begin{matrix} \mathbf{R}_{l} & \mathbf{T}_{l} \\ \mathbf{T}_{l} & \mathbf{R}_{l} \end{matrix} \right]$$

where $\mathbf{H}=i\left( \boldsymbol{\Omega-}\omega\mathbf{I} \right)-\boldsymbol{\Gamma}_{\boldsymbol{e}}-\boldsymbol{\Gamma}_{\boldsymbol{i}}$ and $\mathbf{I}$ is the identity matrix. As demonstrated above, it can be shown that the diagonal block matrices are equal ($\mathbf{R}_{l}\boldsymbol{=}\mathbf{R}_{lf}\boldsymbol{=}\mathbf{R}_{lb}$), and the off-diagonal block matrices are also equal ($\mathbf{T}_{l}\boldsymbol{=}\mathbf{T}_{lf}\boldsymbol{=}\mathbf{T}_{lb}$). Here, the block matrices $\mathbf{R}_{l}$ and $\mathbf{T}_{l}$ are 2 × 2 matrices. Specifically, the matrix $\mathbf{T}_{l}$ represents the non-Hermitian Jones matrix in the linear polarization basis, given by,

$$\mathbf{T}_{l}\mathbf{=}\mathbf{T}_{lu}+\mathbf{T}_{lc}=\xi\mathbf{I}+\eta\left[ \begin{matrix} \Omega_{y}+j\Gamma& K \\ K & \Omega_{x}-j\Gamma\end{matrix} \right].$$

For simplicity, we introduce two dimensionless parameters $\xi=1-\chi/\det\left( \mathbf{H} \right)$ and $\eta=j\gamma_{ex}\gamma_{ey}/\det\left( \mathbf{H} \right)$, where $\chi=\gamma_{ex}\gamma_{ey}+\left\{ \gamma_{ex}\left( \gamma_{iy}-g \right)+\gamma_{ey}\left( \gamma_{ix}-g \right) \right\}/2$. By solving the eigenvalue problem $\mathbf{T}_{l}\left. |\psi_{e} \right\rangle\mathbf{=}t_{e}\left. |\psi_{e} \right\rangle$, the eigenvalues $t_{e}$ (i.e., eigentransmissions) are expressed as:

$$t_{e}=\xi+\eta\left( \frac{\Omega_{x}+\Omega_{y}\pm\sqrt{E}}{2} \right)$$

where ${E=\left( \Omega_{y}-\Omega_{x} \right)}^{2}+4j\Gamma\left( \Omega_{y}-\Omega_{x} \right)+4(K^{2}-\Gamma^{2})$.

The corresponding eigenpolarization states $\left. |\psi_{e} \right\rangle$ (i.e., eigenpolarization states) are proportional to the following vector:

$\left. |\psi_{e} \right\rangle\propto\left[ \begin{matrix} 2K \\ \Omega_{x}-\Omega_{y}-2j\Gamma\pm\sqrt{E} \end{matrix} \right]$.

The requirements for an EP are determined by imposing the following conditions: ${\Omega=\Omega}_{x}=\Omega_{y}, K=\pm\Gamma$ (here, $-$ for LCP and $+$ for RCP). The degenerate eigentransmission is expressed as:

$$t_{e}=\xi+\eta\Omega.$$

The corresponding coalesced eigenvectors are $\left. |\psi_{e} \right\rangle=\frac{1}{\sqrt{2}}\left[ \begin{matrix} 1 & \pm j \end{matrix} \right]^{T}$, where $+$ corresponds to LCP and $-$ corresponds to RCP.

**Derivation of the condition for a chiral zero-eigenvalue at the chiral EP**

The eigentransmission at the chiral EP, $t_{e}=\xi+\eta\Omega$, is equal to the co-polarized transmission coefficients $t_{RR}$ and $t_{LL}$. Therefore, CAPC at the chiral EP is ensured by imposing the conditions $\Omega=0$ and $\xi=0$. To achieve this CAPC condition, the following requirements must be satisfied: $\omega=\omega_{y}=\omega_{x}=\omega_{0}$, $\gamma_{ex}/\gamma_{ey}=-\left( \gamma_{ix}-g \right)/\left( \gamma_{iy}-g \right)=\delta$. When these conditions are met, the non-Hermitian Jones matrix exhibits a chiral EP, resulting in zero eigentransmission and enabling CAPC.

*
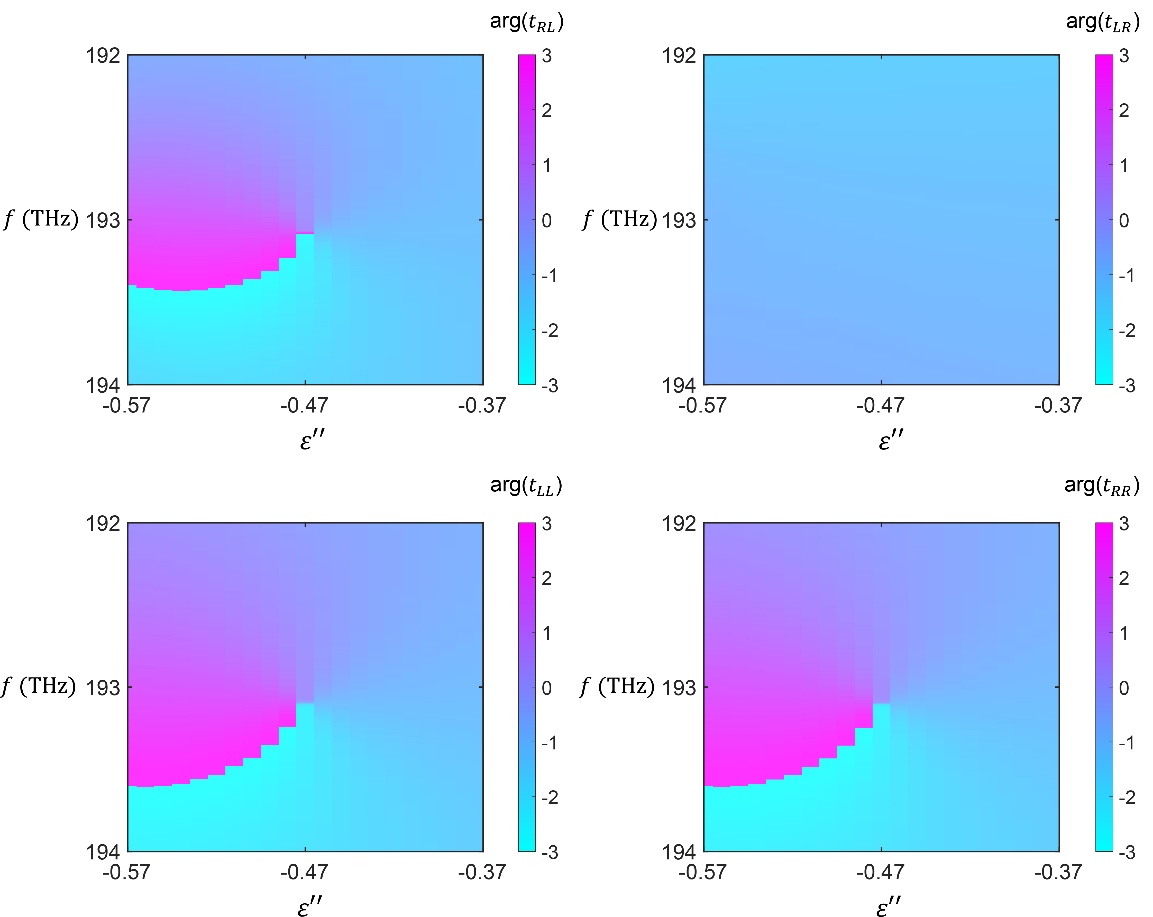
*

**Figure S2.** Transmission phase spectra of $\boldsymbol{t}_{\boldsymbol{RL}}$, $\boldsymbol{t}_{\boldsymbol{LR}}$, $\boldsymbol{t}_{\boldsymbol{LL}}$, and $\boldsymbol{t}_{\boldsymbol{RR}}$ as functions of the imaginary part of the permittivity ε'' and frequency f (THz). The phase distribution reveals winding behavior across this two-parameter space, highlighting the topological nature of the system near the chiral EP.

**Reference**

1. Suh, W., Wang, Z. & Fan, S. Temporal coupled-mode theory and the presence of non-orthogonal modes in lossless multimode cavities. *IEEE J Quantum Electron* **40**, 1511–1518 (2004).

2. Zhao, Z., Guo, C. & Fan, S. Connection of temporal coupled-mode-theory formalisms for a resonant optical system and its time-reversal conjugate. *Phys Rev A  (Coll Park)* **99**, (2019).

3. Kang, M., Chen, J. & Chong, Y. D. Chiral exceptional points in metasurfaces. *Phys Rev A  (Coll Park)* **94**, 1–5 (2016).

4. Li, S. *et al.* Exceptional point in a metal-graphene hybrid metasurface with tunable asymmetric loss. *Opt Express* **28**, 20083 (2020).

5. Baek, S. *et al.* Non-Hermitian chiral degeneracy of gated graphene metasurfaces. *Light Sci Appl* **12**, (2023).

6. Gorkunov, M. V., Antonov, A. A. & Kivshar, Y. S. Metasurfaces with Maximum Chirality Empowered by Bound States in the Continuum. *Phys Rev Lett* **125**, (2020).
